# Lack of effect of citalopram on magnetic resonance spectroscopy measures of glutamate and glutamine in frontal cortex of healthy volunteers

**DOI:** 10.1177/0269881109105679

**Published:** 2010-08

**Authors:** MJ Taylor, R Norbury, S Murphy, S Rudebeck, P Jezzard, PJ Cowen

**Affiliations:** Department of Psychiatry, University of Oxford, Warneford Hospital, Oxford, United Kingdom; Department of Psychiatry, University of Oxford, Warneford Hospital, Oxford, United Kingdom; Department of Psychiatry, University of Oxford, Warneford Hospital, Oxford, United Kingdom; Department of Psychiatry, University of Oxford, Warneford Hospital, Oxford, United Kingdom; The Centre for Functional Magnetic Resonance Imaging of the Brain, University of Oxford, John Radcliffe Hospital, Oxford, United Kingdom; Department of Psychiatry, University of Oxford, Warneford Hospital, Oxford, United Kingdom

**Keywords:** citalopram, frontal cortex, glutamate, magnetic resonance spectroscopy

## Abstract

Magnetic resonance spectroscopy (MRS) is a non-invasive imaging technique that can provide localised measures of brain chemistry *in vivo*. We previously found that healthy volunteers receiving the selective serotonin reuptake inhibitor, citalopram, daily for 1 week showed higher levels of a combined measure of glutamate and glutamine (Glx) in occipital cortex than those receiving placebo. The aim of this study was to assess if a similar effect could be detected in the frontal brain region. Twenty-three healthy volunteers randomised to receive either citalopram 20 mg or a placebo capsule daily for 7–10 days were studied and scanned using a 3T Varian INOVA system before and at the end of treatment. Standard short-TE (echo time) PRESS (Point-resolved spectroscopy) (TE = 26 ms) and PRESS-J spectra were acquired from a single 8-cm^3^ voxel in a frontal region incorporating anterior cingulate cortex. Glutamate and total Glx levels were quantified both relative to creatine and as absolute levels. Relative to placebo, citalopram produced no change in Glx or glutamate alone at the end of the study. Similarly, no effect was seen on other MRS measures studied: myo-inositol, choline, *N*-acetylaspartate and creatine. These data suggest that the effects of serotonin reuptake to modify cortical glutamatergic MRS measures may be regionally specific. This supports the potential for MRS in assessing neuroanatomically specific serotonin-glutamate interactions in the human brain.

## Introduction

Proton magnetic resonance spectroscopy (MRS) is an imaging technique providing safe and non-invasive measurements of aspects of brain chemistry. A range of measures can be obtained including glutamate and the related compound, glutamine. At the field strengths generally available for use in human studies (up to 3 Tesla), reliably separating the MRS signals from glutamate and glutamine is challenging, so the combined level of both glutamate and glutamine (Glx) is often reported (Malhi, *et al*., 2002).

One of the most consistent findings in MRS studies of acute major depressive disorder is lower Glx levels in frontal brain regions (Auer, *et al*., 2000; Hasler, *et al*., 2007; Yildiz-Yesiloglu and Ankerst, 2006). Glutamate released into the extracellular space during neurotransmission is rapidly taken up by astrocytes and converted to glutamine which can be safely transported back to neurons (Danbolt, 2001). This glutamate-glutamine cycle is a major component of brain energetics (Hyder, *et al*., 2006). The reduced levels of Glx suggest some abnormality of this glutamate-glutamine cycle is present during the depressive episodes. Interestingly, it appears that Glx levels in this region return to normal with full clinical recovery (Bhagwagar, *et al*., 2008; Hasler, *et al*., 2005).

Serotonergic agents used in the treatment of depression, such as the selective serotonin reuptake inhibitors (SSRIs), are well placed to modify this glutamate-glutamine cycle, either by actions on neuronal populations to modify the glutamate release or by modulating the astrocyte activity. Serotonergic projections extend throughout cortex (Hornung, 2003), and serotonin receptors are found on both neuronal and astrocyte populations (Eastwood, *et al*., 2001; Jakab and Goldman-Rakic, 1998, 2000).

Recently, we reported that 1 week of citalopram administration at a standard clinical dose was associated with an increase in Glx levels in a posterior cortical region in healthy volunteers (Taylor, *et al*., 2008). The aim of this study was to assess whether a similar finding could be detected in the frontal cortex. In addition, we also used an additional MRS technique, PRESS-J, which permits the measurement of glutamate without glutamine at moderate field strengths (Hurd, *et al*., 2004).

## Materials and methods

### Design

We studied 23 healthy volunteers (11 male, 12 female; mean age 23 years, range 19–32) who were free of any axis I diagnosis assessed using the Standardised Clinical Interview for Diagnostic and Statistical Manual of Mental Disorders-IV (First, *et al*., 1997) and had received no psychoactive medications for at least 3 months before commencing the study. They were also free of any physical illness and taking no medications except the oral contraceptive pill. Participants were randomly assigned to receive either citalopram 20 mg or placebo daily for 7–10 days (the variable time of treatment was necessary to allow for scanner availability). Magnetic resonance imaging (MRI) was performed in the afternoon before starting medication (day 0) and on the day of the final capsule (typically day 7, but day 10 for three participants). Baseline and endpoint mental states and personality traits were assessed by questionnaires (Beck Depression Inventory, Spielberger State Anxiety Inventory and the Positive and Negative Affect Scale).

**Figure 1:**
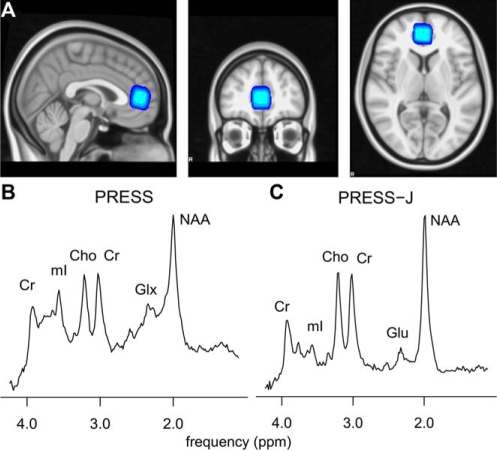
(A) Frontal voxel positioned to include pregenual cingulate cortex (Brodmann areas 24 and 32). (B) Sample PRESS spectrum (echo time 26 ms). (C) Sample PRESS-J spectrum. The PRESS-J spectrum resembles standard PRESS with similar peaks for N-acetylaspartate (NAA), choline (Cho), myo-inositol (mI) and creatine (Cr). However, the Glx doublet is replaced by a singlet glutamate (Glu) peak at 2.35 ppm.

Spectroscopy data were acquired using a 3T Varian INOVA system with a head optimised gradient coil (Tesla Engineering, Storrington, West Sussex, UK) and a head-only transmit/receive quadrature birdcage radiofrequency coil. Data were acquired from a 20 × 20 × 20 mm voxel placed in medial prefrontal cortex anterior to the genu of the corpus callosum (Figure 1). The voxel was positioned manually by reference to an axial *T*_1_-weighted gradient-echo image. PRESS data (Bottomley, 1987) with (TE 26 ms, TR 3 s, averages = 64) and without (TE 26 ms, TR 3 s, averages = 1) water suppressions were acquired. PRESS-J data (Hurd, *et al*., 2004) with and without water suppressions were similarly acquired with TE arrayed from 35 to 195 ms in 10 ms increments (water-suppressed data, total acquisitions = 128; non-water suppressed data, total acquisitions = 16; TR = 3 s). *T*_1_-weighted structural images of whole brain were acquired (2 mm^3^ voxel size). A higher resolution structural image was acquired on a separate occasion using a 1.5 T Siemens Sonata (Siemens, Camberley, UK) scanner using a Turbo FLASH sequence (TR 12 ms, TE 5.65 ms, voxel size = 1 mm^3^).

### MRS analyses

PRESS data were analysed with LCModel (Stephen Provencher Inc., Oakville, Ontario, Canada) (Provencher, 1993), using the non-water suppressed data for eddy current correction, calculating the metabolite concentrations relative to creatine in conventional fashion using 15 metabolite basis spectra and simulated lipid and macromolecule components. PRESS-J data were pre-processed by zero-order phase correction, apodisation with a 5 Hz Gaussian filter and summing of the 16 constituent spectra before analysis. Analysis of the PRESS-J data used Advanced Method for Accurate, Robust, and Efficient Spectral fitting (AMARES) (Vanhamme, *et al*., 1997) since metabolite basis spectra were not available for PRESS-J acquisitions, and the spectral simplification and flat baselines obtained with this technique *in vivo* make direct single peak fitting reliable (Hurd, *et al*., 2004).

### Voxel composition

FMRIB Software Library (University of Oxford, Oxford, UK) FMRIB Automated Segmentation Tool (Zhang, *et al*., 2001) was used to segment the high-resolution structural brain images into grey matter, white matter and cerebrospinal fluid (CSF), to allow estimation of voxel composition.

### Absolute quantitation

The spectroscopy analyses using LCModel and AMARES yielded estimates of concentration relative to creatine. A level referenced to tissue water was also obtained for each measure by correcting for voxel creatine levels (Barker, *et al*., 1993). PRESS-J data were used to estimate both creatine and water levels at *t* = 0, that is, without effects of *T*_2_ decay. Since simply referencing to internal tissue water has been found to underestimate concentrations (Brooks, *et al*., 1999), the values referenced to tissue water were corrected for voxel CSF content and for differences in fractional grey and white matter water density (Lentner, 1981) to provide an estimate of absolute levels. In the absence of an external reference water standard, these are reported in arbitrary units.

### Statistical analyses

Results were analysed using the general linear model with time (pre- and post-treatments) as the within subjects factor and group (placebo vs citalopram) as between subjects factor. For technical reasons, full data sets were not available for four participants. Sensitivity analyses were performed for the effect of including additional factors (gender) and covariates (age, difference in grey matter, white matter and CSF) in the model. Correlations were calculated as Pearson’s product-moment correlation coefficient (*r*^2^). Repeatability coefficients and coefficients of variation for data from the placebo group were calculated. Statistical analyses were performed in R (R Foundation for Statistical Computing, Vienna, Austria) (version 2.5) and SPSS (SPSS Inc., Chicago, IL, USA) (version 15).

## Results

### Participants

Of the 23 participants, 13 received citalopram and 10 received placebo. The groups did not differ in baseline scores of anxiety and depression, days of treatment, or post-treatment mood or anxiety (Table 1).

**Table 1:** Group characteristics

	Citalopram (*n* = 13)	Placebo (*n* = 10)
Age	23 (3)	24 (3)
Gender	5 female, 8 male	7 female, 3 male
Beck Depression Inventory		
Start	1.5 (1.7)	1.8 (2.3)
Endpoint	1.5 (2.1)	1.4 (2.1)
Positive and Negative Affect Scale (positive)		
Start	35.7 (6.9)	37.5 (6.9)
Endpoint	31.0 (7.2)	36.8 (6.8)
Positive and Negative Affect Scale (negative)		
Start	13.7 (6.7)	11.1 (1.6)
Endpoint	13.1 (4.8)	10.2 (0.4)
Spielberger State Anxiety Inventory		
Start	32 (10.9)	28 (8.2)
Endpoint	33 (11.0)	26 (6.5)

Means with standard deviations. No significant differences between groups.

### Voxel characteristics

The spectroscopy data acquired were from a medial prefrontal voxel incorporating pregenual cingulate cortex (Brodmann areas 24 and 32; Figure 1). The voxel composition did not differ between groups or between sessions, and on average, it contained 73% grey matter, 15% white matter and 12% CSF.

### MRS results

**Figure 2:**
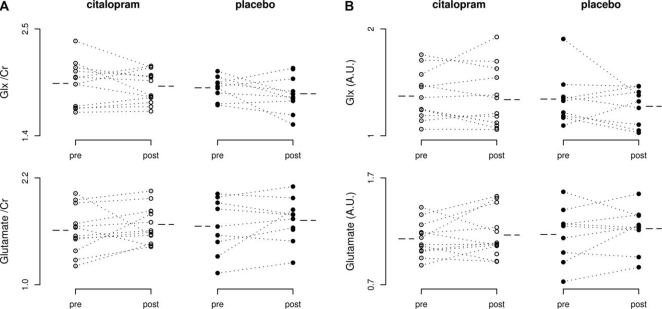
Levels of glutamate + glutamine (Glx) and glutamate, in anterior cingulate cortex before and after 1 week’s administration of citalopram 20 mg daily (*n* = 13, open circles) or placebo (*n* = 10, closed circles) in healthy volunteers. (A) Levels relative to creatine (Cr). (B) Absolute levels in arbitrary units, individual measures (points joined by dotted lines) and group means (dashed lines). No significant group × time interactions observed (Glx/Cr F(1,19) = 0.558, *P* = 0.464; Glu/Cr F(1,19) = 0.063, *P* = 0.805; Glx F(1,17) = 0.002, *P* = 0.964; Glu F(1,17) = 1.122, *P* = 0.304).

There was no significant effect of treatment on any of the MRS measures studied. No main effect of group or time, or group × time interactions were found on analyses of Glx, glutamate, myo-inositol, choline, *N*-acetylaspartate or creatine whether concentrations were expressed relative to creatine or as absolute concentrations (Figure 2).

Measures of Glx and glutamate showed good repeatability across sessions, with mean coefficients of variation of 5.3 and 6.8% for levels relative to creatine and 6.8 and 7.4% referenced to tissue water, with repeatability coefficients from 0.18 to 0.25. Some correlation between Glx and glutamate measures was observed (*r*^2^ = 0.22 with creatine referencing and *r*^2^ = 0.25 with water referencing, *P* < 0.05 in both cases).

### Effect of composition on metabolite levels

For estimates of concentrations relative to creatine, voxel white matter content was correlated with myo-inositol (*r*^2^ = 0.33, *P* < 0.05) and inversely correlated with Glx (*r*^2^ = 0.31, *P* < 0.05). For absolute concentration estimates, the inverse correlation of Glx with white matter content remained (*r*^2^ = 0.4, *P* < 0.05).

## Discussion

The main finding of this study was an absence of effect of 1 week’s citalopram on MRS measures of glutamate and Glx in anterior cingulate cortex in healthy volunteers. This was the case whether concentrations were expressed relative to creatine or as absolute levels.

This lack of effect seems unlikely to be simply explained by the short duration of anti-depressant administration. Although courses of anti-depressant treatment typically last for extended periods, clinical trial data indicate that the beneficial effects of SSRIs relative to placebo in the treatment of depression can be detected after only 1 week (Taylor, *et al*., 2006). Also in healthy volunteers, 1 week of citalopram is associated with consistent changes in emotional processing without overt changes in mood (Harmer, *et al*., 2004), and functional MRI reveals associated changes in neural activity in regions including prefrontal cortex (Harmer, *et al*., 2006). Furthermore, our previous data suggest that the intervention used here increases Glx in occipito-parietal cortex in healthy volunteers (Taylor, *et al*., 2008).

The use of the PRESS-J technique enabled us to measure both glutamate and Glx in this study, but no effect of citalopram on either measure was seen. Glutamine levels were not measured directly, but in the absence of changes in glutamate, altered glutamine would be reflected in changed Glx levels. Therefore, these data suggest that there was no reliable change in glutamine levels. Glx estimates sometimes contain a component attributable to γ-aminobutryic acid (GABA) (Sanacora, *et al*., 2008), and SSRI treatments in healthy subjects can alter GABA levels. However, the magnitude of changes in GABA that might be expected (Bhagwagar, *et al*., 2004; Taylor, *et al*., 2008) would only change Glx estimates by a few percent, which this study lacks the power to detect. Studies using specific sequences to measure GABA would be required to clarify this point (Mescher, *et al*., 1998).

The regional differences, in effect of citalopram, with increased Glx evident in occipital region (Taylor, *et al*., 2008) but not in the anterior cingulate in this study complement a regional pattern of glutamatergic abnormalities in depression and after recovery. Although acute depression is associated with lowered Glx in anterior cingulate cortex (Auer, *et al*., 2000; Hasler, *et al*., 2007; Pfleiderer, *et al*., 2003) and dorsolateral prefrontal cortex (Michael, *et al*., 2003), increased glutamate is reported in occipital cortex (Sanacora, *et al*., 2004). After recovery, increased Glx is found in occipital cortex (Bhagwagar, *et al*., 2007), whereas levels are normalised in anterior cingulate (Bhagwagar, *et al*., 2008; Hasler, *et al*., 2005). Taken together, it is possible that MRS identifies region-specific effects of serotonin reuptake inhibitors on glutamatergic function. The functional significance of this pattern of serotonin-glutamate interactions effects remains to be elucidated.

Finally, it is worth noting that although no change in levels of MRS Glx or glutamate were detected following citalopram treatment in this study, it is certainly possible that citalopram might induce more subtle changes in glutamate-glutamine cycling which would not be detected by the present methodology. For example, proton MRS does not provide a specific measure of synaptic glutamate levels and changes in glutamate release within neurotransmission might be masked by compensatory changes elsewhere. As noted in the Introduction section, the process of glutamate-glutamine cycling is very important in regulating brain neuronal activity and conceivably might be a target for psychotropic drug treatment. However, to study such effects, it will be necessary to use advanced MRS techniques, such as carbon-13 MRS (Shen, *et al*., 1999).
